# Supplementary figures and images for: Developing and validating a dynamic model of water production by direct-contact membrane distillation
Source: PLoS One. 2020 Mar 24;15(3):e0230207. doi: 10.1371/journal.pone.0230207 (PMC7092998; doi:10.1371/journal.pone.0230207)

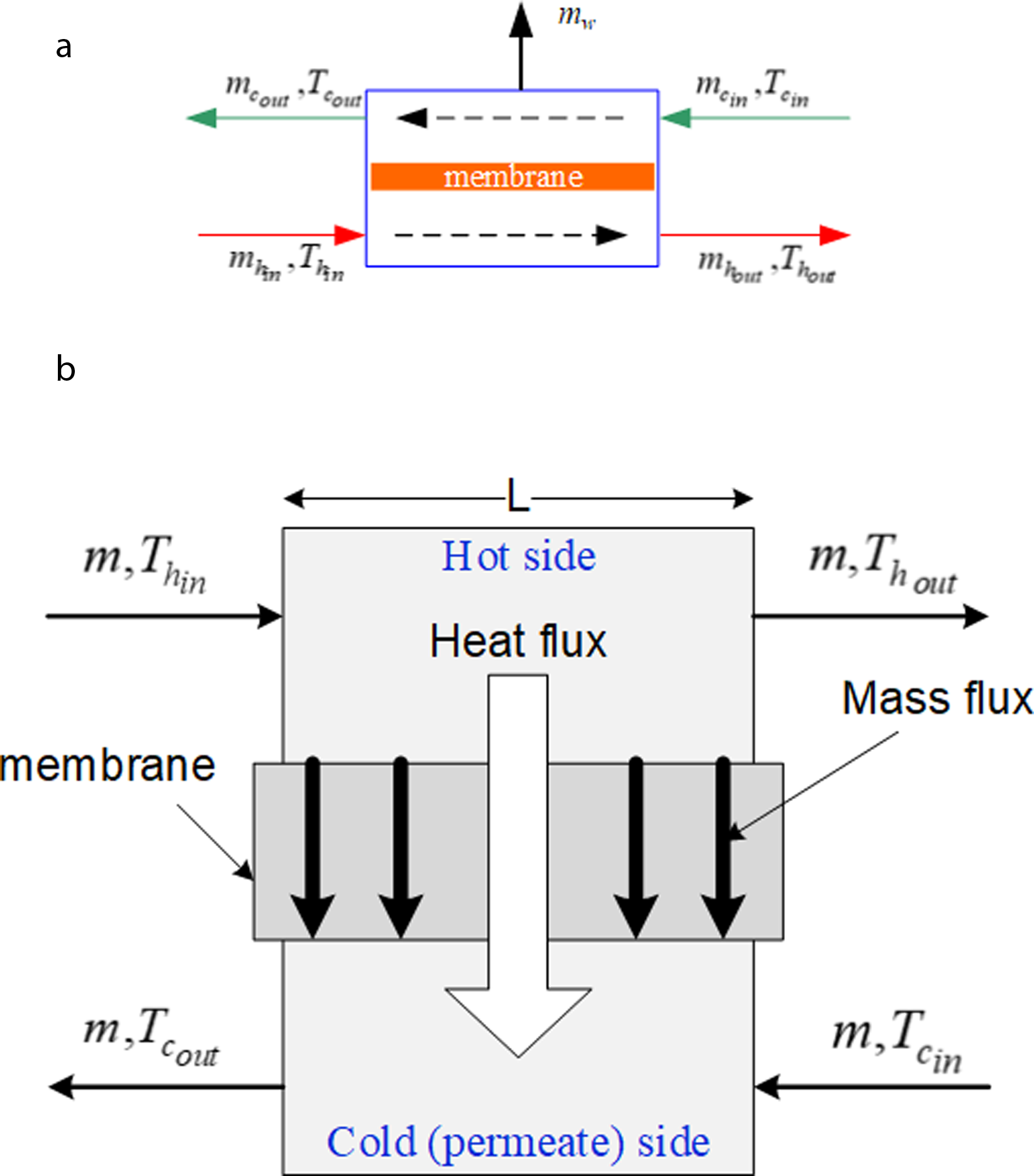

Supplement: S1 Fig — (TIF) [file pone.0230207.s001.tif]
