# Supplementary material for: Developing and validating a dynamic model of water production by direct-contact membrane distillation
Source: PLoS One. 2020 Mar 24;15(3):e0230207. doi: 10.1371/journal.pone.0230207 (PMC7092998; doi:10.1371/journal.pone.0230207)
Supplement: Attachment — Submitted filename: reviewers comments.docx [file pone.0230207.s007.docx]

PONE-D-19-31057
Developing and Validating a Dynamic Model of Water Production in Direct-Contact Membrane Distillation
PLOS ONE

Dear Prof Ali,

Thank you for submitting your manuscript to PLOS ONE. After careful consideration, we feel that it has merit but does not fully meet PLOS ONE’s publication criteria as it currently stands. Therefore, we invite you to submit a revised version of the manuscript that addresses the points raised during the review process.

We would appreciate receiving your revised manuscript by Feb 07 2020 11:59PM. When you are ready to submit your revision, log on to https://www.editorialmanager.com/pone/ and select the 'Submissions Needing Revision' folder to locate your manuscript file.

If you would like to make changes to your financial disclosure, please include your updated statement in your cover letter.

To enhance the reproducibility of your results, we recommend that if applicable you deposit your laboratory protocols in protocols.io, where a protocol can be assigned its own identifier (DOI) such that it can be cited independently in the future. For instructions see: <http://journals.plos.org/plosone/s/submission-guidelines#loc-laboratory-protocols>

Please include the following items when submitting your revised manuscript:

- A rebuttal letter that responds to each point raised by the academic editor and reviewer(s). This letter should be uploaded as separate file and labeled 'Response to Reviewers'.
- A marked-up copy of your manuscript that highlights changes made to the original version. This file should be uploaded as separate file and labeled 'Revised Manuscript with Track Changes'.
- An unmarked version of your revised paper without tracked changes. This file should be uploaded as separate file and labeled 'Manuscript'.

Please note while forming your response, if your article is accepted, you may have the opportunity to make the peer review history publicly available. The record will include editor decision letters (with reviews) and your responses to reviewer comments. If eligible, we will contact you to opt in or out.

We look forward to receiving your revised manuscript.

Kind regards,

Liao Zuwei
Academic Editor
PLOS ONE

Journal Requirements:

1. When submitting your revision, we need you to address these additional requirements.
Please ensure that your manuscript meets PLOS ONE's style requirements, including those for file naming. The PLOS ONE style templates can be found at
http://www.journals.plos.org/plosone/s/file?id=wjVg/PLOSOne_formatting_sample_main_body.pdf and http://www.journals.plos.org/plosone/s/file?id=ba62/PLOSOne_formatting_sample_title_authors_affiliations.pdf

2. We suggest you thoroughly copyedit your manuscript for language usage, spelling, and grammar. If you do not know anyone who can help you do this, you may wish to consider employing a professional scientific editing service.

Whilst you may use any professional scientific editing service of your choice, PLOS has partnered with both American Journal Experts (AJE) and Editage to provide discounted services to PLOS authors. Both organizations have experience helping authors meet PLOS guidelines and can provide language editing, translation, manuscript formatting, and figure formatting to ensure your manuscript meets our submission guidelines. To take advantage of our partnership with AJE, visit the AJE website (<http://learn.aje.com/plos/>) for a 15% discount off AJE services. To take advantage of our partnership with Editage, visit the Editage website (www.editage.com) and enter referral code PLOSEDIT for a 15% discount off Editage services.  If the PLOS editorial team finds any language issues in text that either AJE or Editage has edited, the service provider will re-edit the text for free.

Upon resubmission, please provide the following:

- The name of the colleague or the details of the professional service that edited your manuscript
- A copy of your manuscript showing your changes by either highlighting them or using track changes (uploaded as a *supporting information* file)
- A clean copy of the edited manuscript (uploaded as the new *manuscript* file)

3.  We note that you have stated that you will provide repository information for your data at acceptance. Should your manuscript be accepted for publication, we will hold it until you provide the relevant accession numbers or DOIs necessary to access your data. If you wish to make changes to your Data Availability statement, please describe these changes in your cover letter and we will update your Data Availability statement to reflect the information you provide.

[Note: HTML markup is below. Please do not edit.]

Reviewers' comments:

Reviewer's Responses to Questions

**Comments to the Author**

1. Is the manuscript technically sound, and do the data support the conclusions?

The manuscript must describe a technically sound piece of scientific research with data that supports the conclusions. Experiments must have been conducted rigorously, with appropriate controls, replication, and sample sizes. The conclusions must be drawn appropriately based on the data presented.

Reviewer #1: Partly

Reviewer #2: Yes

2. Has the statistical analysis been performed appropriately and rigorously?

Reviewer #1: No

Reviewer #2: Yes

3. Have the authors made all data underlying the findings in their manuscript fully available?

The [PLOS Data policy](http://www.plosone.org/static/policies.action#sharing) requires authors to make all data underlying the findings described in their manuscript fully available without restriction, with rare exception (please refer to the Data Availability Statement in the manuscript PDF file). The data should be provided as part of the manuscript or its supporting information, or deposited to a public repository. For example, in addition to summary statistics, the data points behind means, medians and variance measures should be available. If there are restrictions on publicly sharing data—e.g. participant privacy or use of data from a third party—those must be specified.

Reviewer #1: Yes

Reviewer #2: Yes

4. Is the manuscript presented in an intelligible fashion and written in standard English?

PLOS ONE does not copyedit accepted manuscripts, so the language in submitted articles must be clear, correct, and unambiguous. Any typographical or grammatical errors should be corrected at revision, so please note any specific errors here.

Reviewer #1: No

Reviewer #2: No

5. Review Comments to the Author

Please use the space provided to explain your answers to the questions above. You may also include additional comments for the author, including concerns about dual publication, research ethics, or publication ethics. (Please upload your review as an attachment if it exceeds 20,000 characters)

**Reviewer #1**:

1. The Experimental devices were same as the reference [32,35,38,39], what’s the main difference between this paper and the reference [32,35,38,39]. The innovations should be clarified.
*Answer*: new statements were added in line 117-121 to elaborate this issue.

2. Adjustment of the model by adding heat loss term resulted in enhanced predictions, but the adjustment is also based on regression and fitting. So the universalities of the modified model need to be discussed. And there were no theoretical explanations. The Experimental devices were a MD pilot plant, and the scale-up problems are unavoidable when the MD is applied in practical industries, then the adjustment may not be applicable. And carrying out experiments in practical industries is not realistic.
*Answer*: we agree that the model losses its predictiveness with such modification. That is why we strove to formulate the heat losses as function of the process parameter and minimized its tuning parameter to a single variable, flos. Moreover, variation of flos with flow rate remains reasonable. Nevertheless, our aim is not only calibrating the model, but also to highlight shortcomings of the process design and/or models such that engineers and researchers seek remedies to facilitate the industrialization of MD technology.

3. The Figures are not clear enough to be published. Legends were missed in some Figures.

*Answer*: Regarding Figure resolution, all figures have passed the PACE test provided in the Web site of the PLOSone Journal. Legends of Figure 2,3,9 and 10 were placed in one subplot to avoid overcrowding the plot. Nevertheless, we reinserted the legends in all figure as required by the reviewer. Note for figurer 7 & 8 the legends are inserted in the figure caption because the space in the plot area is very limited.

**Reviewer #2:**

1. The experimental results and analysis did not clearly indicate the advantages and significance of the spatial model constructed in this manuscript.

*Answer*: In fact, the spatial model showed superior performance compared to the standard lumped model as manifested by the relative error given in Table 1. But it provided comparable or slightly inferior performance when modified lumped model is used, i.e. when averaged bulk temperature is imposed (Eq. A.24). In this case, the lumped bulk temperatures (Eq. A.24) became smaller than the average of the spatial bulk temperature over the membrane length creating smaller ΔT and hence mass flux. Moreover, the lumped bulk temperature became less sensitive to modelling uncertainty as the average bulk temperature is weighted by the inlet fixed temperature, i.e. ${T_{h}}_{in}, {T_{c}}_{in}$. This is evident because when tuning is incorporated, both models provided similar results because the modelling uncertainty is compensated by the tuning method.

2. It is recommended to mark the legends in each Figure to make the physical meaning of each line more intuitive.

*Answer*: Legends are inserted for all figures and their subplots. Exception is figure 7 and 8 where the legends are written in the figure caption because the space in the plot area is very limited.

2. In general, there is a lack of explanation of the lumped model slightly better than the spatial model.

*Answer*: this issue is discussed above.

4. The overall idea of the article is clear, but the appendix is relatively long. It is recommended to include part of the appendix in the main text.

*Answer*: Appendix A lists the step by step derivation of the dynamic model, therefore, we recommend keeping the entire derivation together for better follow up. Hence it can not be apportioned and moved to the main text.

5. It is noted that your manuscript needs careful editing by someone with expertise in technical English editing paying particular attention to English grammar, spelling and sentence structure so that the goals and result of the study are clear to the reader.

*Answer*: the manuscript is edited by the English editing services provided by King Saud University.

6. PLOS authors have the option to publish the peer review history of their article ([what does this mean?](https://journals.plos.org/plosone/s/editorial-and-peer-review-process#loc-peer-review-history)). If published, this will include your full peer review and any attached files.


If you choose “no”, your identity will remain anonymous but your review may still be made public.


**Do you want your identity to be public for this peer review?** For information about this choice, including consent withdrawal, please see our [Privacy Policy](https://www.plos.org/privacy-policy).

Reviewer #1: No

Reviewer #2: No


[NOTE: If reviewer comments were submitted as an attachment file, they will be attached to this email and accessible via the submission site. Please log into your account, locate the manuscript record, and check for the action link "View Attachments". If this link does not appear, there are no attachment files to be viewed.]


While revising your submission, please upload your figure files to the Preflight Analysis and Conversion Engine (PACE) digital diagnostic tool, <https://pacev2.apexcovantage.com/>. PACE helps ensure that figures meet PLOS requirements. To use PACE, you must first register as a user. Registration is free. Then, login and navigate to the UPLOAD tab, where you will find detailed instructions on how to use the tool. If you encounter any issues or have any questions when using PACE, please email us at [figures@plos.org](mailto:figures@plos.org). Please note that Supporting Information files do not need this step.
